# System biology and bioinformatics pipeline to identify comorbidities risk association: Neurodegenerative disorder case study

**DOI:** 10.1371/journal.pone.0250660

**Published:** 2021-05-06

**Authors:** Utpala Nanda Chowdhury, Shamim Ahmad, M. Babul Islam, Salem A. Alyami, Julian M. W. Quinn, Valsamma Eapen, Mohammad Ali Moni

**Affiliations:** 1 Department of Computer Science and Engineering, University of Rajshahi, Rajshahi, Bangladesh; 2 Department of Electrical and Electronic Engineering, University of Rajshahi, Rajshahi, Bangladesh; 3 Department of Mathematics and Statistics, Imam Mohammad Ibn Saud Islamic University, Riyadh, Saudi Arabia; 4 Healthy Ageing Theme, Garvan Institute of Medical Research, Darlinghurst, NSW, Australia; 5 School of Psychiatry, Faculty of Medicine, University of New South Wales, Sydney, Australia; 6 WHO Collaborating Centre on eHealth, School of Public Health and Community Medicine, Faculty of Medicine, UNSW Sydney, Sydney, Australia; University College London Institute of Neurology, UNITED KINGDOM

## Abstract

Alzheimer’s disease (AD) is the commonest progressive neurodegenerative condition in humans, and is currently incurable. A wide spectrum of comorbidities, including other neurodegenerative diseases, are frequently associated with AD. How AD interacts with those comorbidities can be examined by analysing gene expression patterns in affected tissues using bioinformatics tools. We surveyed public data repositories for available gene expression data on tissue from AD subjects and from people affected by neurodegenerative diseases that are often found as comorbidities with AD. We then utilized large set of gene expression data, cell-related data and other public resources through an analytical process to identify functional disease links. This process incorporated gene set enrichment analysis and utilized semantic similarity to give proximity measures. We identified genes with abnormal expressions that were common to AD and its comorbidities, as well as shared gene ontology terms and molecular pathways. Our methodological pipeline was implemented in the R platform as an open-source package and available at the following link: https://github.com/unchowdhury/AD_comorbidity. The pipeline was thus able to identify factors and pathways that may constitute functional links between AD and these common comorbidities by which they affect each others development and progression. This pipeline can also be useful to identify key pathological factors and therapeutic targets for other diseases and disease interactions.

## Introduction

Alzheimer’s disease (AD) is the most frequent neurodegenerative disease (NDD) which is considered to be the current primary cause of dementia, causing most of all dementia cases (60% to 80%). 5,700,000 Americans are estimated to have AD in 2018, and this number is projected to reach 13.8 million by 2050 [1]. It was a major cause of mortality in 2015, 110,561 deaths from AD were officially recorded in that year in the United States [1]. The main features of AD include cognitive deficiency including memory loss and diminished abilities to carry out simple everyday activities [2], in addition to depression, apathy, hallucinations, delusions and aggression [3]. Significant AD-related features seen in the central nervous system include localized accumulations of beta-amyloid (A*β*) protein in plaques in the extracellular space and tau protein tangles inside neurons. Whether these are primary causes or pathophysiological responses to AD are unclear, but these features (and by implication the AD pathogenic processes) can be present over 20 years before AD cognitive symptoms become clearly evident. The pathogenic mechanisms that underlie AD initiation and development are very poorly understood, although a number of genetic and environmental risk factors have been associated with AD [4, 5]. The apolipoprotein E (APOE4) is evidenced to be related to AD throughout the world population [6–8]. Genetic studies suggest that less than one percent of AD cases arise due to genetic mutations involving the amyloid precursor protein (APP) and the presenilin 1 and presenilin 2 protein-related genes that give rise to plaques [9]. Nevertheless, the inheritance of APP or presenilin 1 gene mutants is associated with a high probability for AD development, consistent with an important role for their corresponding proteins [10]. To this day, no disease modifying drugs for AD are available, all the FDA approved drugs only alleviate the symptoms. Most of the clinical trials for AD-therapeutics are A*β*-based and they have failed [11].

Symptoms of other NDDs become evident at any point during the course of AD development. Moreover, AD and some other NDDs share similar genetic and environmental risk factors indicating their possible coexistence. Parkinson’s disease (PD) is the second-most common NDDs after AD, characterized by the deficiency of striatal dopamine due to the neuronal loss in the substantia nigra, along with deposition of *α*-synuclein in neurons [12–14]. Neuronal death and neural dysfunction caused by oxidative stress and mitochondrial DNA (mtDNA) variants are reported to be associated with both AD and PD [15, 16]. Huntington’s disease (HD) is usually an inherited and autosomal dominant disorder that causes brain cell damage [17]. Neuropathologic characteristics of PD, HD and AD are evidenced to be consistent that involves neurotoxins in their pathogenesis [18]. Amyotrophic lateral sclerosis (ALS) is a lethal NDD that triggers decay of motor neurons and eventually control of the motor system is lost [19]. ALS and dementia share genetic sensitivity resulting in their co-occurrences [20]. The TNF*α*-signaling axis and neuroinflammation, both play a significant role in the pathogenesis of ALS and AD [21]. Spinal Muscular Atrophy (SMA) is mostly an inherited NDD with autosomal recessive nature. Both HD and SMA are entirely monogenic conditions caused by a mutation in the huntingtin gene (HTT) [22] and the SMN1 gene [23] respectively. Lewy Body Disease (LBD) is the primary cause of dementia after AD, particularly in aged people [24]. The cognitive impairments resulted in both LBD and AD are directly associated with the synaptic loss [25, 26]. *α*-synuclein is found to have a notable influence in the pathogenesis of LBD and AD [27]. Frontotemporal dementia (FTD) is a focal variety of dementia associated with the continuous deterioration surrounding the prefrontal and anterior temporal cortex [28]. FTD and AD patients show identical executive functions which indicate similar abnormalities in the frontal lobes [29]. Multiple sclerosis (MS) is an inflammatory disease that affects the brain and spinal cord, and results in intellectual trouble [30]. The central nervous system of MS and AD patients exhibit a key contribution of the microglia activation [31]. Therefore, the cognition impairment in AD highly influences the progression and presentation of other NDDs.

However, inadequate understanding of AD and its consequences, that means how these NDDs and AD influence each other is unknown [32]. Such co-occurrences can be investigated at a molecular level, for example by identifying genes with altered expression or molecular pathways that are shared by the NDDs and AD [33]. Previously developed data analysis methods for disease comorbidity studies include comoR [34], POGO [35], CytoCom [36], comoRbidity [37] and Comorbidity4j packages [38]. comoR, POGO and comoRbidity are R packages where the first one maps disease comorbidity leveraging patient diagnosis, gene expression and clinical data. POGO predicts comorbidity risk using multiple omics analysis approaches with, ontology and phenotype data. comoRbidity, on the other hand, integrates clinical data along with genotype-phenotype information for comprehensive comorbidity analysis. CytoCom is a Cytoscape App for disease comorbidity network visualization. Finally, Comorbidity4j is an open-source Java-based web-platform that uses clinical information to identify a group of comorbidity indices and thus provides significant disease comorbidity. However, the use of gene expression analyses in the study of comorbidity may offer improved insights into AD disease mechanisms [39]. The availability of huge public transcriptomics resources such as microarray data and bioinformatics tools has enabled us to perform comorbidity analyses, i.e., identify gene pathways that enable two diseases to influence each other [40, 41]. This study aims to take advantage of the transcriptomics data to demonstrate how AD and other NDDs impact each other at the molecular level through a series of bioinformatics and computational approaches.

## Materials and methods

### Data

We obtained gene expression datasets from the National Center for Biotechnology Information (NCBI) Gene Expression Omnibus (GEO) and European Bioinformatics Institute Array Express database. We queried for AD and found 531 datasets, most of them were disqualified at the start by being very low sample size compared to our selected cut off sample size 10, duplicate datasets, having inappropriate format or undesirable experimental set-up, RNAseq datasets, and from organisms other than human. Thus we selected 8 datasets to be highly relevant to AD and appropriate for our study. The finally selected gene expression datasets for AD have the accession numbers: GSE1297, GSE110226, GSE33000, GSE48350, GSE12685, GSE5281, GSE4229 and GSE4226. All datasets were generated using central nervous system tissues and Affymetrix array platforms except GSE4226 and GSE4229 which were MGC arrays of peripheral blood analyses. GSE1297 is a correlation analysis of hippocampal tissues from nine control subjects and 22 AD patients with varying severity [42]. GSE110226 compared transcripts of choroid plexus from postmortem tissues of 6 healthy samples and 7 AD patients, 4 FTD patients and 3 HD patients [43]. GSE33000 analysed post mortem prefrontal cortex tissues of 310 AD patients, 157 HD patients and 157 non-demented samples [44]. GSE48350 is the profiling of hippocampus, entorhinal cortex, superior frontal cortex and post-central gyrus regions in 170 healthy individuals and 80 AD cases [45]. GSE12685 is a comparative study of gene expression for frontal cortex synaptoneurosomes between 6 normal controls and 8 AD patients [46]. GSE5281 is obtained by analyzing 16 unaffected and 19 AD affected tissues, specifically 6 central nervous system tissues: entorhinal cortex, hippocampus, medial temporal gyrus, posterior cingulate, superior frontal gyrus and primary visual cortex cells [47]. GSE4229 is a study of genetic variations of peripheral blood mononuclear cells from 22 healthy old people and 18 AD cases using the NIA Human MGC cDNA microarray [48]. GSE4226 compares peripheral blood mononuclear cells obtained from 14 normal elderly control (NEC) and 14 AD affected subjects [49]. For the study of neurodegenerative comorbidity analysis of AD we selected GSE7621, GSE6613, GSE49036 and GSE54536 for PD; GSE93767, GSE110226 and GSE33000 for HD; GSE833 and GSE107375 for ALS; GSE27206 for SMA; GSE49036 for LBD; GSE110226, GSE13162 and GSE40378 for FTD; GSE21942 for MS. GSE7621 is generated by extracting RNA from substantia nigra tissue of postmortem brain of 9 controls and 16 PD patients and hybridizing on Affymetrix microarrays [50]. GSE6613 is whole blood expression data analysis from PD patients and controls [51]. GSE49036 is an overall study of gene expression of subtantia niagra tissue from PD patients, LBD cases and normal individuals [52]. GSE54536 is obtained through a whole-transcriptome comparison of the peripheral blood from PD patients with healthy subjects [53]. GSE93767 is a transcriptional analysis of human-induced pluripotent stem cells (hiPSC) using a CRISPR-Cas9 from HD cases compared with controls [54]. GSE833 is a gene expression profiling of grey matter from post mortem spinal cord of ALS patients and controls [55]. GSE107375 is a whole transcriptome expression analysis of the motor cortex from 10 controls and 30 ALS cases [56]. GSE27206 is the gene expression data evaluation of induced pluripotent stem cells (iPS cells) for SMA [57]. GSE13162 is obtained through global expression profiling using a microarray of postmortem brain cells from the frontal cortex, hippocampus, and cerebellum [58]. GSE40378 is a gene expression analysis by an array of induced pluripotent stem cell models [59]. GSE21942 is a comparison of the expression level of genes for peripheral blood mononuclear cells between MS patients and controls [60].

### Gene ontologies

The gene ontology (GO) is a uniform illustration of gene and gene product attributes for all organisms. This project aims to model a biological system starting from the molecular level and expanding towards pathway, cellular and organism-level systems [61]. Among the three categories of GO, we incorporated the biological process (BP) for annotation in this study. The disease ontology (DO) project, on the other hand, represents comprehensive information about inherited, developmental and acquired human diseases using open-source ontology [62]. The DO terms used in this study for the corresponding diseases are AD DOID: 10652, PD DOID: 14330, HD DOID: 12858, ALS DOID: 332, SMA DOID: 12377, LBD DOID: 12217, FTD DOID: 9255 and MS DOID: 2377.

### Gene set enrichment analysis

Gene set enrichment analysis (GSEA) is the procedure of identifying differentially expressed genes (DEGs) in a large set of genes, that may be correlated with disease phenotypes [63]. It uses a set of statistical methods to group genes considering the commonality in their expression level, biological process or chromosomal position. This is done by comparing the expression pattern in disease condition and healthy state. These genes may be acquired using DNA microarray or next-generation sequencing (NGS). The genes having a decisive level of expression are picked up as DEGs (both over and under-expressed).

### Semantic similarity

Semantic similarity is a measure of similarity between terms (DEGs, GO, DO) using ontologies by estimating a topological closeness [64]. This method uses directed acyclic graphs (DAGs) to compute the information contented by each terms considering statistical annotations. The exact position of these terms in the DAG and the connection with their predecessor terms determines the semantic measure. An ontology term *T* can be denoted by the DAGs *DAG*_*T*_ = (*T*, *A*_*T*_, *E*_*T*_), where *A*_*T*_ is a set of ancestor terms of *T* and *E*_*T*_ is a set of edges connecting the terms in *DAG*_*T*_ that represent the semantic relation. At first, the semantic measure of each term is represented numerically as,

$$\{\begin{array}{ll} S_{T}(T)=1 & \text{t}=\text{T}\\ S_{T}(t)=max\{w_{e}*S_{T}(t\text{'})|t\prime \in \text{decendants}\;\text{of}\;(t)\} & \text{t}\neq T \end{array}$$ (1)

Here *t* is a general term, *t*′ a descendant term and *w*_*e*_ the semantic participation of *t* with *t*′. The inclusive semantic measure for *T* is

$$\begin{array}{c} SM\left(T\right)=\sum_{t\in A_{T}}S_{T}\left(t\right) \end{array}$$ (2)

Now, if *DAG*_*X*_ = (*X*, *A*_*X*_, *E*_*X*_) and *DAG*_*Y*_ = (*Y*, *A*_*Y*_, *E*_*Y*_) are two terms *X* and *Y* respectively, then their semantic similarity is

$$\begin{array}{c} sem\_sim\left(X,Y\right)=\frac{\sum_{t\in T_{X}\cap T_{Y}}\left[S_{X}\left(t\right)+S_{Y}\left(t\right)\right]}{SM(X)+SM(Y)} \end{array}$$ (3)

Given two sets of terms *T*_1_ = {*t*_11_, *t*_12_, ….*t*_1*l*_} and *T*_2_ = {*t*_21_, *t*_22_, ….*t*_2*m*_} having lengths *l* and *m* respectively, the semantic similarity the term sets *T*_1_ and *T*_2_ is

$$sem\_sim_{BMA}(T_{1},T_{2})=\frac{\sum_{i=1}^{l}max_{1\leq j\leq m}sem\_sim(t_{1i},t_{2j})+\sum_{j=1}^{m}max_{1\leq i\leq l}sem\_sim(t_{1i},t_{2j})}{l+m}$$ (4)

with *i*, *j* indices on *T*_1_, *T*_2_ terms.

### Overview of the analytical process

At first, the chosen gene expression datasets and their matrix information were downloaded and converted to Expression Set class for differential gene expression analysis. We reviewed the sample records (GSM) manually for sample classification and constructed design models (patients, controls). The created design model for AD cases is AD patient vs healthy individual and patient of neurodegenerative diseases vs healthy control for other cases. These design models are then filtered using a linear and a Bayesian method. Using a threshold for p-value and absolute log Fold Change (logFC) values to be at most 0.05 and at least 1.0 respectively, DEGs are identified.

**Fig 1:**
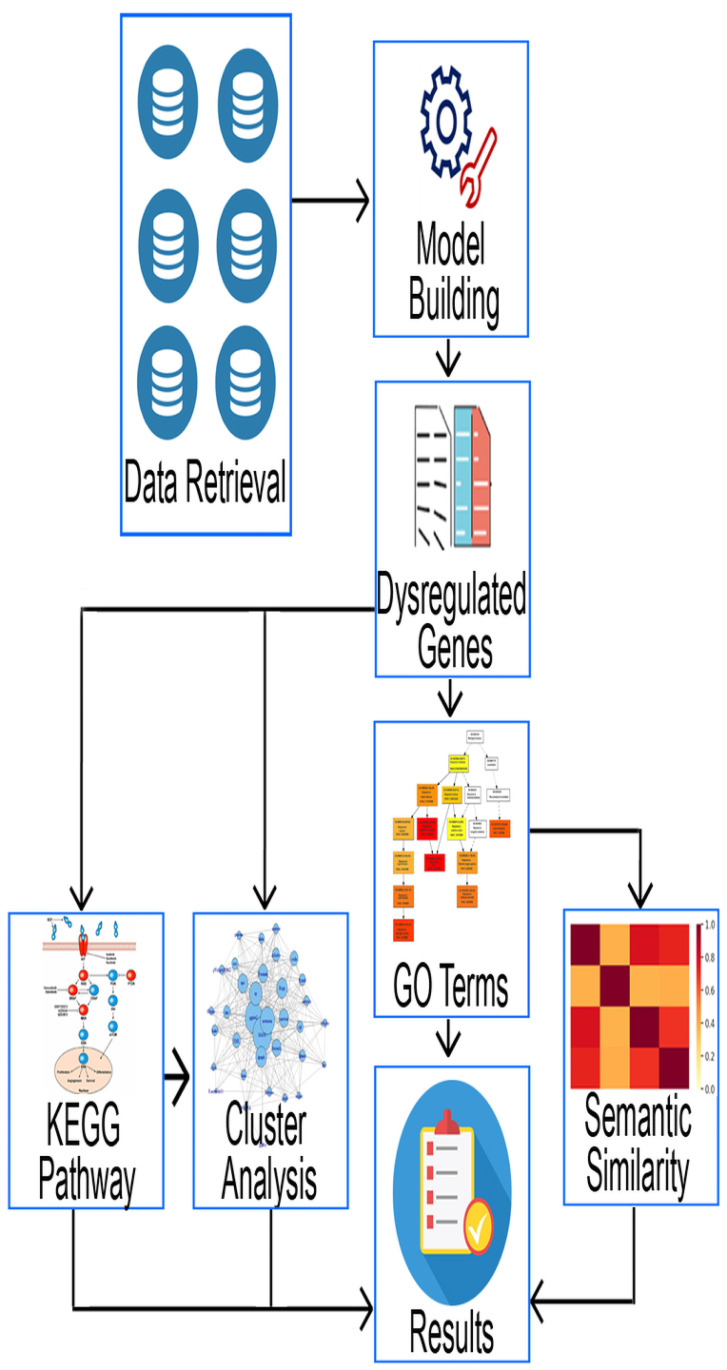
Pipeline of the analytical approach.

We constructed the topGOdata class using the selected genes by specifying the GO domain and stipulating the annotation to perform the mapping. We then obtained the filter for GO terms and their associations with the DEGs by employing the Fisher’s exact test. After that, we performed the semantic similarity comparison among all the selected diseases considering DEGs, GO terms and DO terms to measure the proximity for all the chosen datasets. We then performed the KEGG pathway [65] analysis for the DEGs to find out significant molecular pathways or diseases for AD and its comorbidity datasets. Finally, the statistical information, genes-GO term associations, DAGs, semantic similarity measures along with dendrograms for DEGs, GO terms and DO terms are generated as final output. Furthermore, we generated a gene network using the common DEGs between AD and its comorbidities, with enlightenment on the pathways/diseases. Fig 1 pictures the block diagram of the analytical process.

The implementation of the analytical approach is divided into two main R scripts, that are available at: https://github.com/unchowdhury/AD_comorbidity. Various BioConductor 3.4 R packages [66] were used to develop the analytical approach. We downloaded the selected datasets from the NCBI GEO and converted the data into form Expression Set class using GEOquery 2.40.0. GEOquery offers corresponding methods to access various types of GEO data [67]. Linear Models for Microarray Data (limma) 3.30.8 was used for differential gene expression analysis by comparing the transcriptomic profiles of healthy subjects with that of the patients. Limma provides compact collection of tools to analyze gene expression microarray data [68]. We filtered the genes using genefilter 1.56 for the threshold values p-value less than 0.05 and absolute logFC greater than 1. Genefilter offers necessary methods to curate genes obtained in high throughput experiments [69]. We incorporated the topGO 2.26 for the enrichment analysis for GO and performed the Fisher’s exact test to obtain the topology of the DAG [70]. The semantic similarity between the selected pathologies were determined for GO terms and DEGs using GOSemSim 2.0.4 that serves as a quantitative tool for the semantic comparisons [71]. The semantic similarity for DO terms was evaluated by Disease Ontology Semantic and Enrichment analysis (DOSE) 3.0.10 [72]. Finally, the KEGG pathway enrichment analysis was performed using clusterProfiler 3.2.14, which offers statistical analysis and visualization methods for functional profiles of genes [73]. We used the GEO file transfer protocol (ftp) call to download GEO datasets instead of using GEOquery package due to some interaction issues with other used packages.

## Results

### Statistical summary and GO term trees

The statistics about all the chosen AD studies are mentioned in Table 1. The threshold for p-values is 0.05 and for absolute logFC is 1.0 to obtain the number of genes shown in 4th, 5th and 6th columns from left. The numbers shown in brackets for 6th column are obtained using 2.0 as threshold values of logFC. Similarly, Table 2 summarizes the statistics for the selected neurodegenerative comorbid pathologies of AD. Table 3 shows the synopsis of the selected datasets along with the number of analyzed DEG.

**Table 1:** Statistical summary for AD studies.

Dataset	Tissue source	Genes	P-Value	Adj. P-Value	LogFC	GO Terms	Fisher test
GSE110226	Choroid plexus	21003	6002	475	442 (24)	200	11
GSE12685	Frontal cortex synaptoneurosomes	13907	2986	1	180 (0)	211	26
GSE1297	Hippocampal CA1 Tissue	13907	2830	0	565 (10)	156	9
GSE33000	Prefrontal cortex	19518	16105	15858	0 (0)	201	26
GSE4226	Peripheral blood mononuclear	6571	457	0	581 (299)	84	21
GSE4229	Peripheral blood mononuclear	6571	332	0	432 (219)	135	6
GSE48350a	Hippocampus	22832	10222	3515	322 (9)	147	14
GSE48350b	Entorhinal cortex	22832	7002	645	114 (6)	197	7
GSE48350c	Superior frontal cortex	22832	8419	2537	78 (6)	125	6
GSE48350d	Post-central gyrus	22832	5416	435	21 (5)	84	4
GSE5281	Entorhinal cortex, hippocampus, medial temporal gyrus, posterior cingulate, superior frontal gyrus and primary visual cortex	22832	12726	10699	2306 (35)	113	18

The 3rd, 4th, 5th and 6th columns represent the number of unfiltered genes, the number of significant DEGs with threshold for p-value, adjusted p-value and logFC (numbers in brackets are for logFC with threshold 2) respectively. 7th and 8th columns show the number of unfiltered GO terms and significant GO terms considering Fisher exact test.

**Table 2:** Statistical summary for studies of neurodegenerative comorbid diseases of AD.

Dataset	Dis.	Tissue source	Genes	P-Value	Adj. P-Value	LogFC	GO Terms	Fisher test
GSE49036	PD	Substantia nigra	22832	6454	67	228 (3)	249	25
GSE6613	PD	Whole blood	13907	1991	0	4 (0)	106	6
GSE7621	PD	Substantia nigra	22787	4389	1	1672 (55)	102	19
GSE54536	PD	Peripheral blood	20760	8466	5855	4009 (1631)	64	22
GSE110226	HD	Choroid plexus	21003	3542	1	313 (12)	76	30
GSE33000	HD	Prefrontal cortex	19518	16328	16144	0 (0)	112	14
GSE93767	HD	Induced pluripotent stem	20053	1245	2	1632 (92)	61	11
GSE49036	LBD	Substantia nigra	22832	3651	0	184 (3)	100	19
GSE68605	ALS	Motor neurons	22832	2596	7	5768 (343)	404	49
GSE833	ALS	Spinal cord	6068	765	19	2555 (931)	343	56
GSE110226	FTD	Choroid plexus	21003	5164	0	629 (29)	77	25
GSE13162	FTD	Frontal cortex, hippocampus, and cerebellum	13907	4771	2099	139 (1)	43	15
GSE40378	FTD	Induced pluripotent stem	20760	3752	565	21 (2)	43	15
GSE21942	MS	Peripheral blood	22832	9379	5876	524 (62)	84	25
GSE27206	SMA	Induced pluripotent stem	22832	2117	0	1225 (232)	99	43

The 4th, 5th, 6th and 7th columns represent the number of unfiltered genes, the number of significant DEGs with threshold for p-value, adjusted p-value and logFC (numbers in brackets are for logFC with threshold 2) respectively. The 8th and 9th columns show the number of unfiltered GO terms and significant GO terms considering Fisher exact test.

**Table 3:** Summary of findings in the steps of the pipeline for the datasets of the selected pathologies.

Disease	Tissue source	Available dataset	Selected dataset	Up DEGs	Down DEGs
Alzheimer’s Disease	Brain, blood	531	8	2037	1598
Parkinson’s Disease	Brain, blood	196	4	961	1345
Huntington’s Disease	Brain	64	3	315	418
Lewy Body Disease	Brain	11	1	57	93
Amyotrophic Lateral Sclerosis	Brain, spinal cord	104	2	1563	1666
Frontotemporal Dementia	Brain	28	3	447	278
Multiple Sclerosis	Blood	124	1	213	317
Spinal Muscular Atrophy	Brain	20	1	250	211

**Fig 2:**
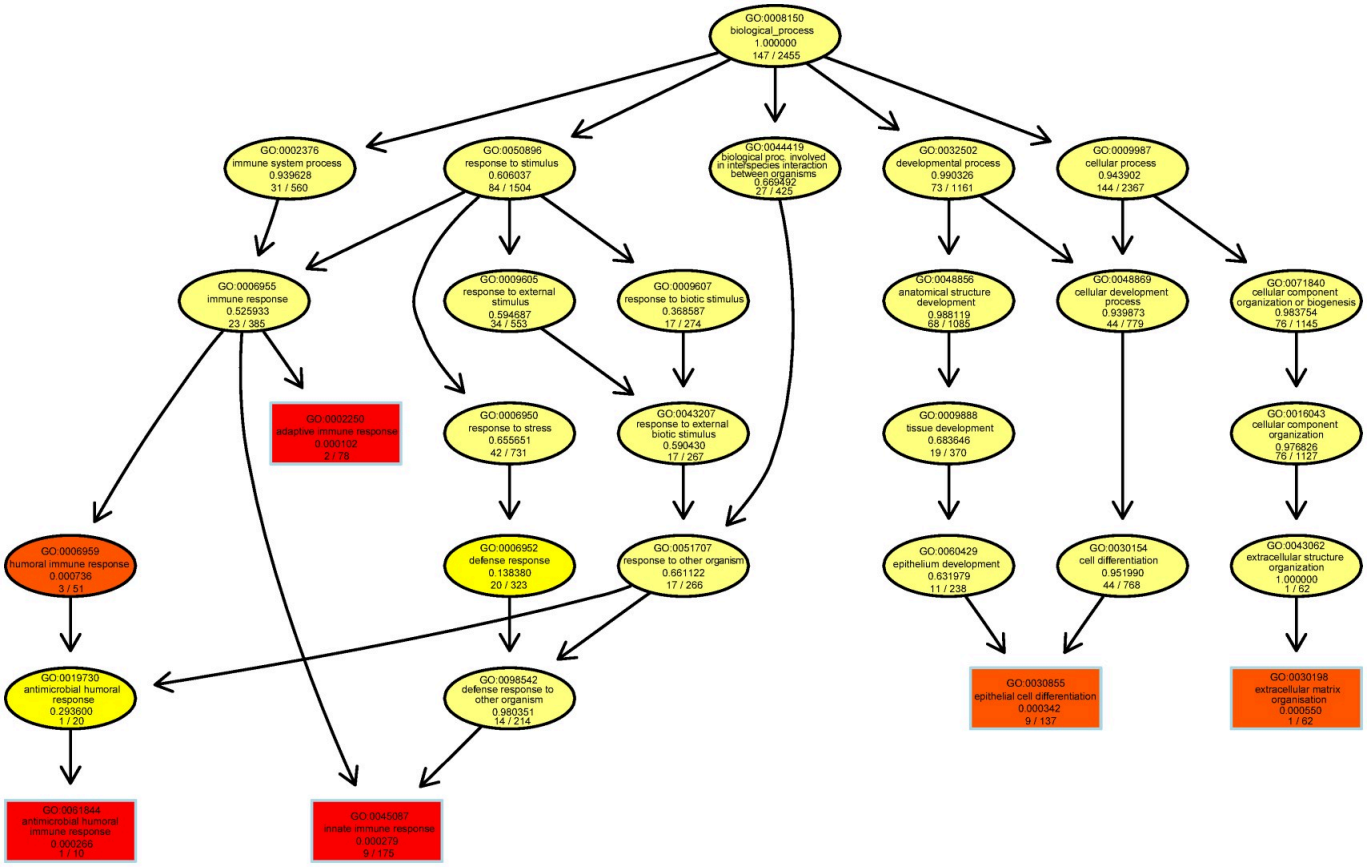
Example DAG of GO terms with GSEA on GSE12685 dataset of AD. The original graph (on the top) and a zoom (on the bottom) are presented. The 5 most significantly enriched GO terms are indicated by the rectangles and the oval shaped nodes represent significant GO terms. The red and orange colors indicate the most significant GO terms. The last two lines inside each node show raw p-value followed by the number of significant genes and the total number of genes annotated to the corresponding GO term for the dataset.

DAG of GO terms is constructed for each selected pathologies. The graphs manifest that all the GO terms are not trivial and hence are hidden. Fig 2 shows such a DAG for the dataset GSE12685 of AD study.

### Pathways

The five most significant BP GO terms involved in each AD study are as follows:

i GSE110226: immune system process, regulation of immune system process, positive regulation of immune system process, nitrogen compound metabolic process, and transport.
ii GSE12685: adaptive immune response, antimicrobial humoral immune response, innate immune response, epithelial cell differentiation and extracellular matrix organisation.
iii GSE1297: immune system process, nitrogen compound metabolic process, cell communication, system process, and transport.
iv GSE33000: biological process, nitrogen compound metabolic process, signal transduction, cell communication, and transport.
v GSE4226: reproduction, cell activation, regulation of cell growth, response to active oxygen species and response to the acid chemical.
vi GSE4229: biological process, metabolic process, nitrogen compound metabolic process, cell communication and signal transduction.
vii GSE48350a: biological process, cellular process, nitrogen compound metabolic process, metabolic process and transport.
viii GSE48350b: nitrogen compound metabolic process, cell communication, system process, response to stress and transport.
ix GSE48350c: biological process, cellular process, metabolic process, regulation of biological process and regulation of the cellular process.
x GSE48350d: cell activation, myeloid leukocyte activation, myeloid cell activation involved in immune response, endothelial cell activation involved in immune response, cell activation involved in immune response and immune effector process.
xi GSE5281: nitrogen compound metabolic process, response to stress, cellular aromatic compound metabolic process, nucleobase-containing compound metabolic process and transport.

**Fig 3:**
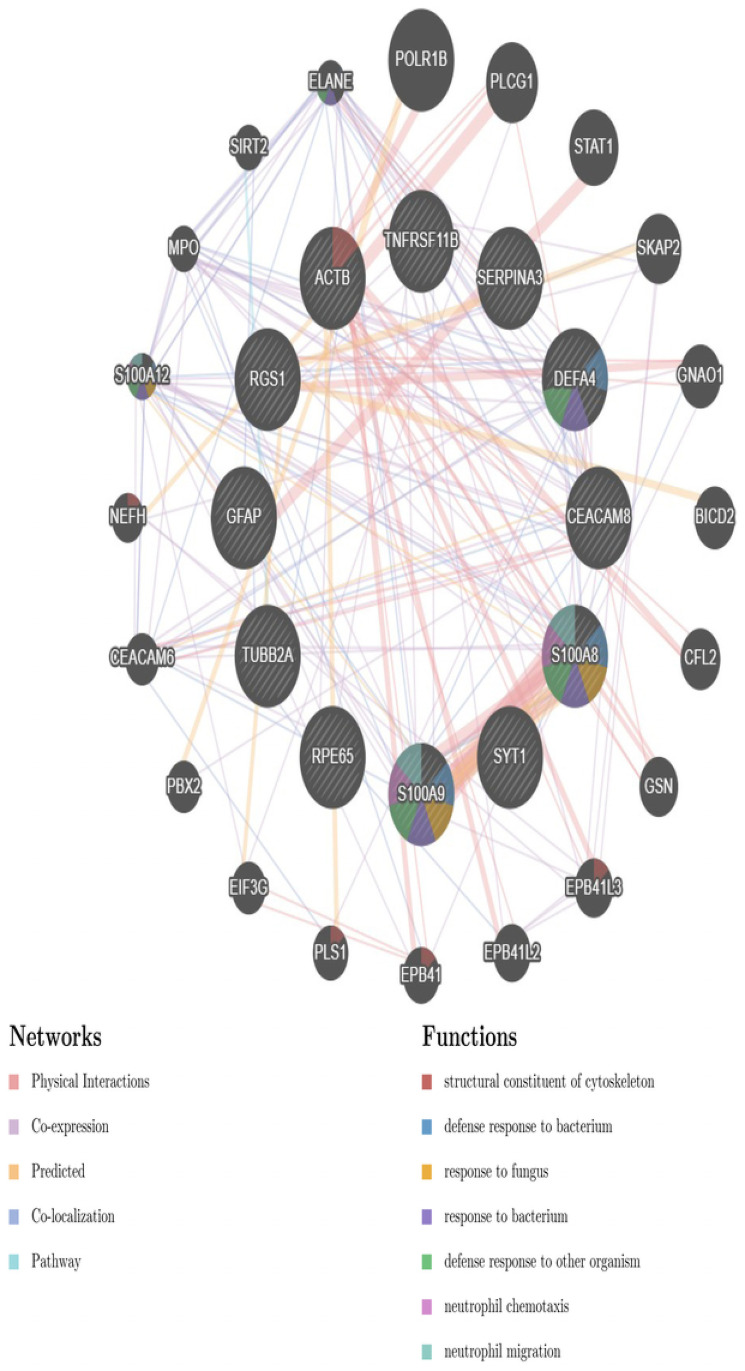
Cluster network with overlapping DEGs between AD and other selected pathologies obtained using the online tool GeneMania [74]. Nodes indicate DEGs and links represent functional associations. The node size indicates the rank of the gene considering its association with other nodes and width of the edges represent the percentile contribution of the connecting nodes to a particular functional association.

The DEGs comparison between the AD datasets and its neurodegenerative comorbidities reveals the following overlapping genes: ACTB, CEACAM8, COX2, DEFA4, GFAP, MALAT1, RGS1, RPE65, SYT1, S100A8, S100A9, SERPINA3, TNFRSF11B and TUBB2A. We built a cluster network for these overlapping DEGs using the online tool GeneMania [74]. For this we took physical interactions, co-expression, predicted, co-localization and pathway into consideration. The network shown in Fig 3 indicates 32 related genes (nodes) and 183 links between them. The most significant pathways associated with the chosen pathologies and their percentile contributions are a structural constituent of the cytoskeleton (7.35%), defense response to a bacterium (6.58%), response to fungus (27.27%), response to a bacterium (2.99%), defense response to other organisms (2.66%), neutrophil chemotaxis (8.33%), neutrophil migration (8.33%), chemokine production (6.82%), regulation of inflammatory response (2.84%) and inflammatory response (1.77%).

### Semantic similarity and KEGG enrichment

**Fig 4:**
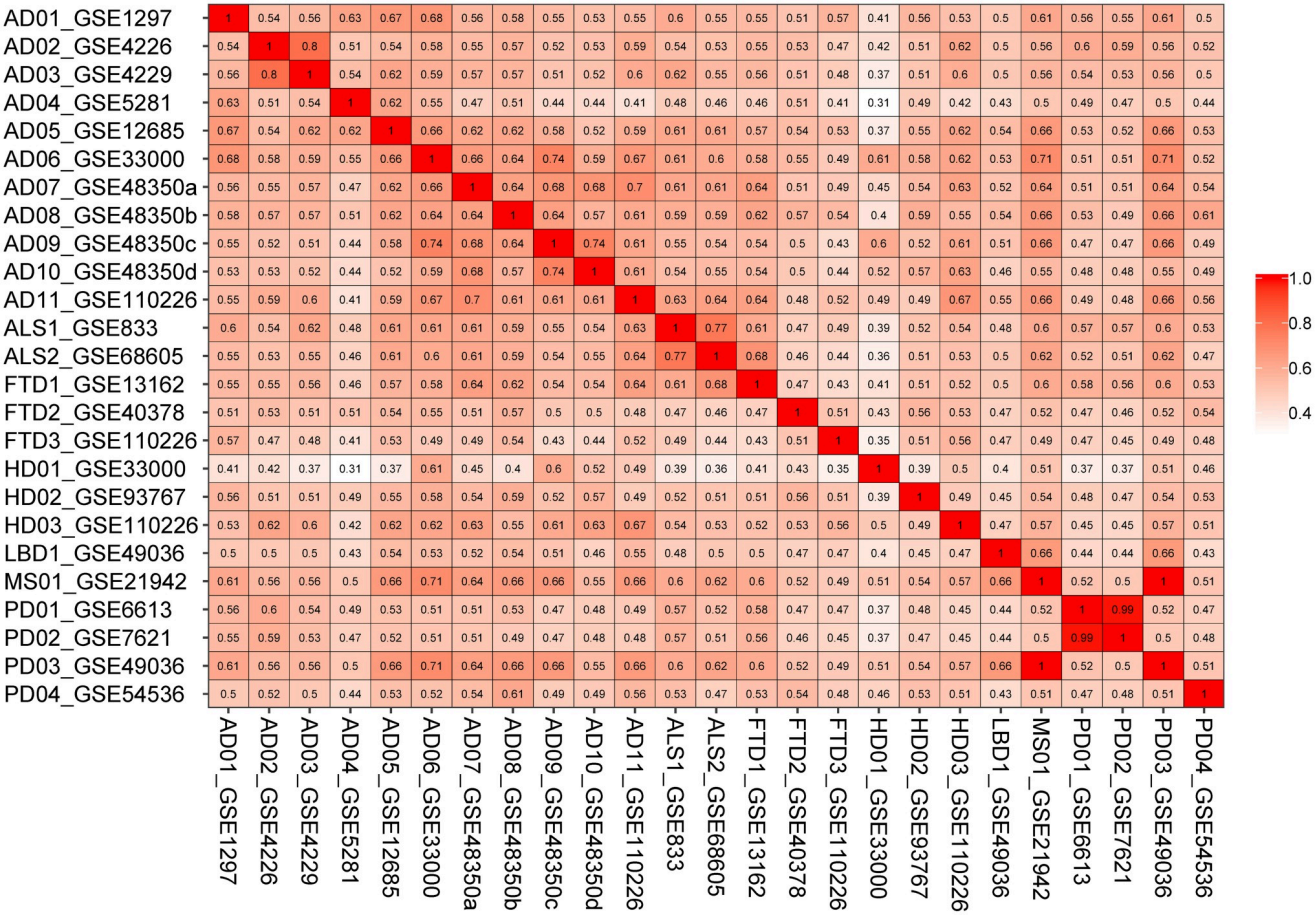
Semantic similarity matrix for the differential expressed genes in the five most significant GO terms. The first two letters of each entry represents the selected pathologies (AD-Alzheimer’s disease, ALS-Amyotrophic lateral sclerosis, FTD-Frontotemporal dementia, HD-Huntington’s disease, LBD-Lewy body disease, MS-Multiple sclerosis, PD-Parkinson’s disease).

The semantic similarity measures for DEGs of the selected disease conditions are represented in a matrix as shown in Fig 4. AD06_GSE33000 is associated with two selected comorbidities: Parkinson’s disease and multiple sclerosis exhibiting the value of semantic similarity at least 0.7. Considering other evidence from AD11_GSE110226 and AD07_GSE48350a/b, Parkinson’s disease, Huntington’s disease, amyotrophic lateral sclerosis, frontotemporal dementia, multiple sclerosis and spinal muscular atrophy are closely associated with AD.

**Fig 5:**
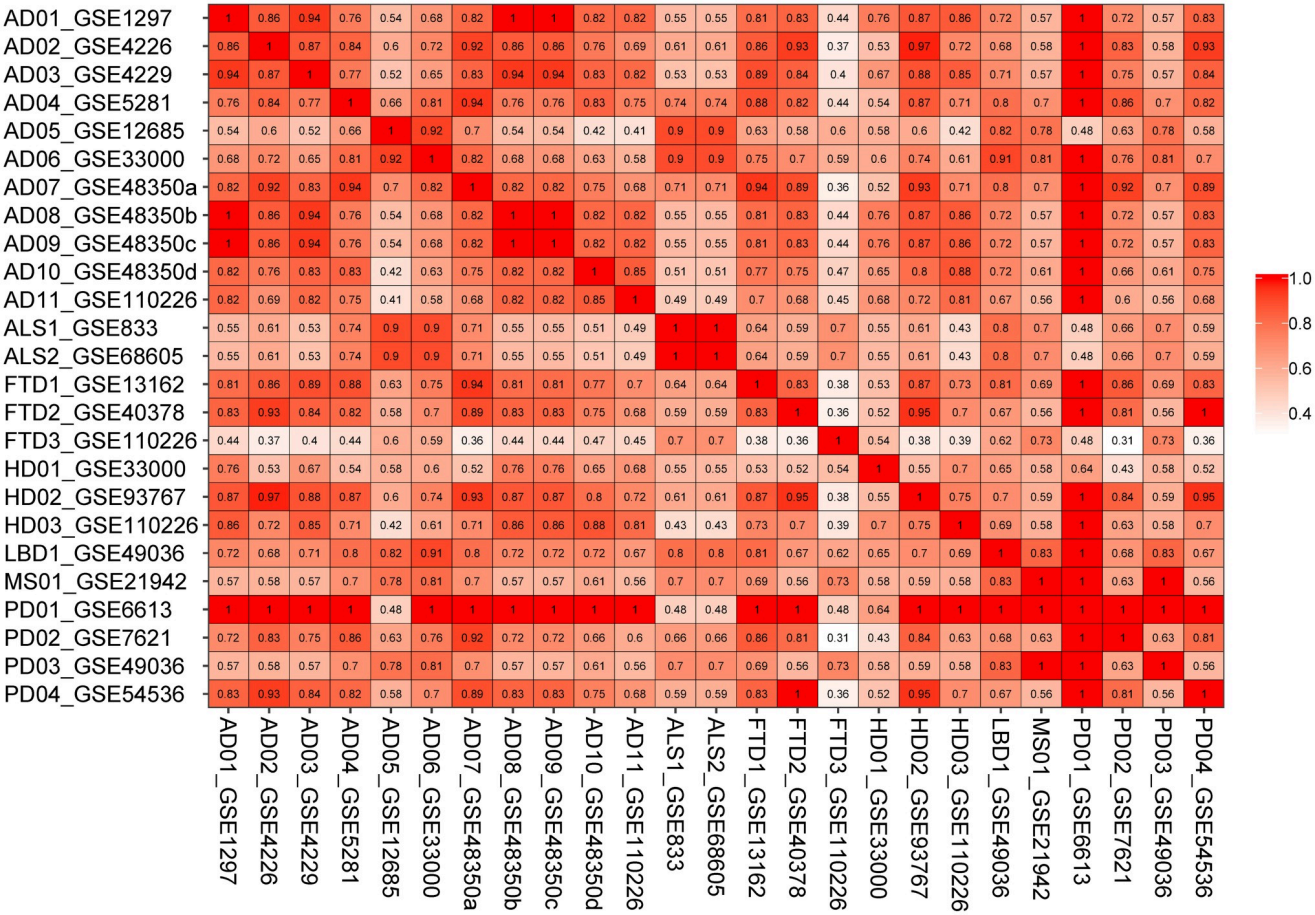
Semantic similarity matrix for the five most significant GO terms. Entry names are similar as Fig 4.

Fig 5 depicts the semantic similarity matrix for the top five GO terms. Notably, all AD datasets except AD05_GSE12685 are similar (semantic similarity value of 1) to PD01_GSE6613 dataset considering the top five GO terms. In addition, observing the semantic similarity measure being greater than 0.9, AD05_GSE12685 and AD06_GSE33000 are well clustered with both amyotrophic lateral sclerosis datasets. But if we inspect the semantic similarity measure at least 0.8, all Parkinson’s disease, Huntington’s disease, Lewy body disease, amyotrophic lateral sclerosis, frontotemporal dementia, multiple sclerosis and spinal muscular atrophy employs significant similarity with some of the AD datasets.

**Fig 6:**
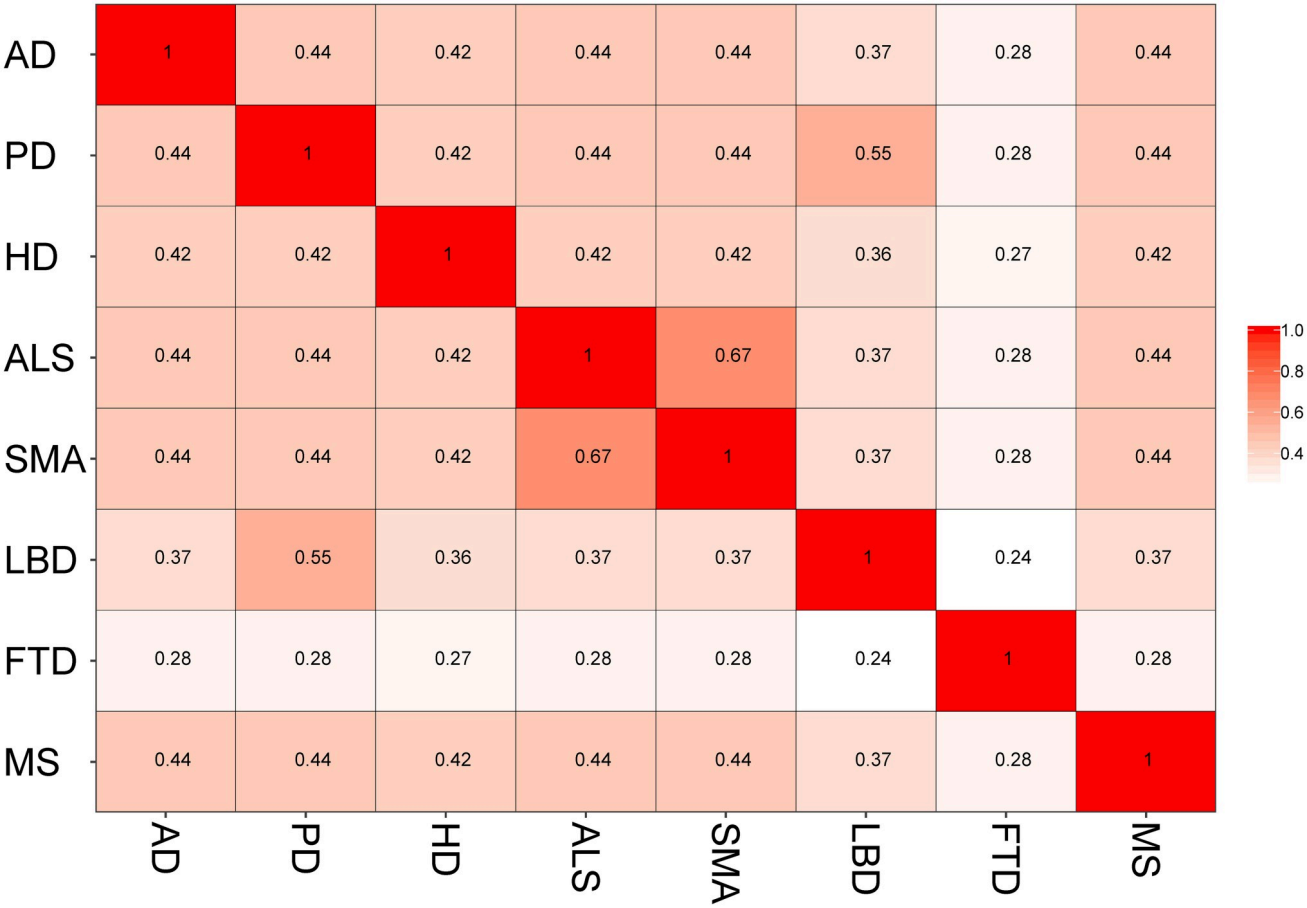
Semantic similarity matrix for DO terms. AD-Alzheimer’s disease, ALS-Amyotrophic lateral sclerosis, FTD-Frontotemporal dementia, HD-Huntington’s disease, LBD-Lewy body disease, MS-Multiple sclerosis, PD-Parkinson’s disease.

Fig 6 represents the matrix of DO terms using semantic similarity. Surprisingly, AD exhibited very trivial association with other NDDs considering the DO terms analysis data. Notable significance was observed between spinal muscular atrophy and amyotrophic lateral sclerosis (0.67). On the other hand, Parkinson’s disease showed significant association (0.55) with lewy body disorder.

**Fig 7:**
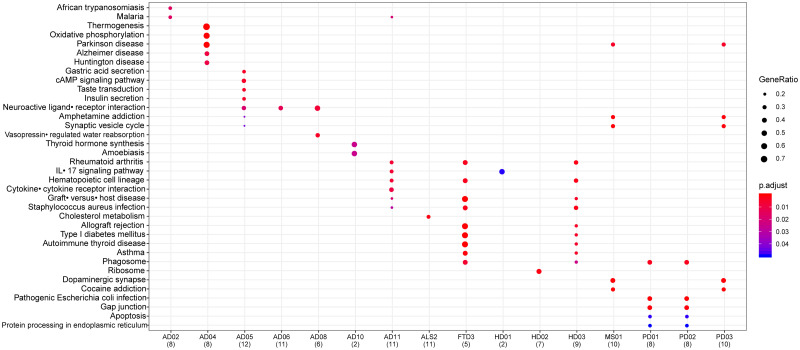
KEGG pathway enrichment analysis for differentially expressed genes. Each row represents a KEGG pathway associated with the diseases shown in columns. The domination of genes in the pathway indicated by the dimension of the circles and the range of the circles represents the statistical validation for p-value = 0.05.

Fig 7 shows the KEGG pathway association with all selected datasets. Resulting pathways with at least two occurrences among AD datasets are neuroactive ligand-receptor interaction and malaria. Moreover, recurring pathways common between at least one AD dataset and other pathologies are Parkinson’s disease, amphetamine addiction, synaptic vesicle cycle, rheumatoid arthritis, hematopoietic cell lineage, graft-versus-host disease, Staphylococcus aureus infection and IL-17 signaling pathway.

## Discussion

In this work, we introduced an analytical framework of bioinformatics analysis for AD-comorbidity studies and demonstrated its efficacy for mining information in public databases. We employed this approach on AD and other NDDs using selected microarray gene expression data from public databases. We applied GSEA to DEGs that we identified, and identified related molecular pathways and their association among selected transcriptomic data using GO and DO. Moreover, we also investigated the effectiveness of semantic similarity as a proximity measure between the diseases using selected ontologies. Identification of the interconnection within a set of pathologies at the molecular level can certainly enrich our insight about the disease mechanism and eventually promotes the possibility for accurate diagnosis and efficacious remedy planning. Our approach leverages publicly available gene expression data from microarray experiments ensuring the possibility of reusing available data. This yields an opportunity to extract hidden information from previously published and publicly accessible datasets. Furthermore, we considered data from different sources and also for different cell types to demonstrate the robustness of the work. Utilization of patient omics data is opening new windows for enhancement in clinical decision making including disease risk assessment, accurate diagnosis and subtyping, treatment planning and dose determination [75]. Incorporation of such data into patient care by medical practitioners through clinical activities such as electronic prescribing of medications is a serious prospect. In the near future, aspects of both personalized and preventive medicine will become clinically feasible with potential disease progression assessed by tracking multiple layers of omics and clinical data from healthy individuals. Our work provides methodologies for comorbidity analysis and enhanced visualization as an effective analytical approach that can help professional physicians.

Among the obtained overlapping genes, GFAP has been reported to be associated with AD [76], ALS [77] and MS [78]. Analyzing the co-occurrence of GO terms and molecular pathways between AD and its comorbid neurodegenerative diseases several significant terms and pathways were found to be common. Defects of Oxidative phosphorylation has clear association with AD and PD [79, 80]. Upregulation in cAMP signaling pathway has implication with AD [81]. The association of neuroactive ligand-receptor interaction with *α*-synuclein is involved in PD [82]. IL-17 signaling pathway has been reported to be involved in the pathogenesis of chronic neuroinflammatory disorder like AD, MS, FTD and HD [83, 84]. The dopaminergic system contributes in neuromodulation and hence the dopaminergic synapse pathways evoke the onset and progression of disorders of central nervous system [85]. The gap junctions connect the cytoplasm of adjacent cells and such interconnections in central nervous system cells maintain normal function. Gap junctions are involved in the pathology of most neurological diseases [86].

We carried out analytical processes for AD and common neurodegenerative comorbidities, although this can be employed for any other AD datasets with other comorbidities if the datasets contain adequate samples for both diseases affected cases and healthy controls. We selected the cutoff sample size 10 considering at least five individuals with active disease state and at least five healthy samples. Our methodology is implemented in an R programming platform that incorporates several other packages from the Bioconductor repository, although these can be easily substituted with another implementation using a different platform. From the methodological point of view, such approaches have been successfully demonstrated various disease interactions recently [41, 87]. It’s noteworthy, however, that the dataset selection would have some qualitative and quantitative effects on the outcomes. The findings documented here could be enhanced by incorporating more datasets from other sources as well as different cell types. Nevertheless, our study has employed a new and innovative analytical approach for comorbidity analysis of these complex diseases.

## Conclusion

We investigated how the methodology described in this manuscript can be used to analyse the transcriptome of AD and neurodegenerative diseases that are common comorbidities; we employed techniques of interconnected processes, inflammation pathways, associations of different omics data in terms of different ontology, such as GO and DO. This has two advantages: a better insight into AD composing comorbidity disease networks and the presentation of a novel pipeline constituting statistical analysis for complex diseases. Moreover, the neurodegenerative disease comorbidity analysis of AD presented here could be utilized for improving diagnosis and to help the discovery of novel therapeutic targets. Therefore, our methodology and pipeline could move forward the clinical decision making for personalized medicine.
